# Nitric Oxide is a Potential Diagnostic Marker for Hepatocellular Carcinoma

**DOI:** 10.3797/scipharm.1307-09

**Published:** 2013-08-08

**Authors:** Laila A. Eissa, Nada H. Eisa, Mohamed A. Ebrahim, Maha Ragab, Amal M. El-Gayar

**Affiliations:** 1 Department of Biochemistry, Faculty of Pharmacy, Mansoura University, Mansoura, 35516, Egypt.; 2 Oncology Center, Mansoura University, Mansoura, 35516, Egypt.; 3 Department of Internal Medicine, Faculty of Medicine, Mansoura University, Mansoura, 35516, Egypt.

**Keywords:** Nitric oxide, Glutathione reductase, Hepatocellular carcinoma, Alpha-fetoprotein

## Abstract

Hepatocellular carcinoma (HCC) is the fifth most common cancer in men and the seventh most common in women. This cancer varies widely in incidence throughout the world, with rising incidence in Egypt. HCC is considered the second most frequent cause of cancer incidence and mortality among men in Egypt. This study aimed to estimate the serum levels of nitric oxide (NO) and glutathione reductase in order to evaluate their role as oxidative status markers in HCC development and progression. For this purpose, serum levels of these parameters were assessed in 50 HCC patients, and 30 cirrhotic patients in addition to 15 healthy subjects as a control group. In the present study, glutathione reductase activity showed a significant increase in HCC as compared to the control group (P= 0.019). On the other hand, no significant difference was observed between the cirrhotic and HCC patients (P= 0.492). Serum NO was significantly higher in patients with HCC than in cirrhotic patients (P= 0.001) or the control group (P= 0.001), with a sensitivity of (74%) and specificity of (88.89%) at a cut-off level of 614.1 μmol/l. While AFP, alpha-fetoprotein, at a cutoff level of 200 ng/ml had a sensitivity of (52%), the specificity was (100%). Indeed, nitric oxide was high in 62.5% of AFP-negative HCC patients. In conclusion, glutathione reductase has no role in HCC diagnosis. However, nitric oxide is a potential diagnostic marker for HCC. The simultaneous determination of serum nitric oxide and AFP gave significant improvement in the detection of HCC patients compared to that of AFP alone.

## Introduction

Hepatocellular carcinoma (HCC) is the fifth most common cancer in men and the seventh most common in women [1]. The incidence of this particular cancer varies throughout the world, with rising incidence in Egypt [2]. In Egypt, HCC was considered as a common malignant tumor, accounting for about 4.7% of chronic liver disease patients with most HCC patients being presented at a late stage in approximately 85% of cases [3].

Detection of HCC at early stages is critical for good clinical outcomes as the prognosis of HCC patients is very poor when diagnosed at late stages. Although serum AFP is the most established tumor marker in HCC and considered as the gold standard to which other markers are compared, it was found to be normal in about 30% of the patients, especially in early stages [4]. Ultrasonography is an important tool for the diagnosis of HCC, however it depends on the operator’s experience, and accordingly the validity of other biomarkers in the diagnosis of HCC needs to be investigated [5].

Nitric oxide (NO) plays an important role in HCC development and its progression. During the pre-HCC period, viral infection (hepatitis B and C) or other unforeseen circumstances may lead to uncontrolled, prolonged, and/or massive production of NO by inducible nitric oxide synthase (iNOS) in the liver [6]. At the same time, NO released by the tumor cells might enhance angiogenesis, which can lead to accelerated growth of the primary tumor, as well as facilitate the process of metastasis [6].

The glutathione antioxidant system in cirrhosis and hepatocellular carcinoma is imbalanced and supports the hypothesis that oxidative stress plays an important role in the development of those liver diseases [7]. Glutathione reductase is highly specific for the oxidized form of glutathione (GSSG) and could be used in the detection of liver injury [8].

Thus, we assessed the diagnostic accuracy of nitric oxide and glutathione reductase activity as biochemical markers of HCC. Added to this we assessed the sensitivity and specificity of nitric oxide as a marker for HCC diagnosis.

## Experimental

### Subjects

In the present study, we measured serum nitric oxide and glutathione reductase levels in patients with HCC, cirrhotic patients, and healthy controls and evaluated a possible diagnostic value using these markers. From March 2012 to September 2012, 50 patients with HCC (37 males and 13 females; aged 40–90 years with a mean ± SE of 60.7 ± 1.29) were recruited from the Oncology Center, Mansoura University, Mansoura, Egypt. Since all HCC patients in this study have cirrhosis as an underlying liver disorder and in order to nullify the effect of cirrhosis on the level of the studied parameters, a group of 30 cirrhotic patients, (19 males and 11 females; aged 33–80 years with a mean ± SE of 56.4 ± 1.6), without any evidence of HCC was used and selected from the outpatient clinic of the Specialized Medical Hospital, Mansoura University, Mansoura, Egypt. All cases involved in this study were tested for either pathological proof or a typical radiologic pattern on the post-contrast study plus the diagnostic serum AFP. The severity of liver disease was assessed by the Child–Pugh classification [9]. The stage and management were defined according to the Barcelona-Clinic Liver Cancer Group diagnostic and treatment strategy (BCLC) [10]. Patients with other types of malignancy, advanced organ failure, and advanced medical co-morbidity were excluded from the study. A control group comprised of 15 healthy individuals (11 males and 4 females; aged 51–63 years with a mean ± SE of 55.7 ± 1.24) with no apparent evidence of active disease or medical disorders was selected.

### Blood Sampling

Fasting blood samples were collected from all patients and control groups and were subsequently divided into two portions. The first portion was collected in tubes containing ethylenediaminetetraacetic acid (EDTA) and used for blood picture investigation within 5 hours. The second portion was collected in a monovette without additives. This blood was left to clot for 20–30 min at room temperature, followed by centrifugation at 1500 rpm for 10 min. The serum was then transferred to a polypropylene tube and if the analysis was not performed immediately, the samples were frozen and maintained at −80°C until use.

### Analysis of Biochemical Parameters

Serum α-fetoprotein was measured using a commercially available ELISA kit from (DiaMetra Company). The serum nitric oxide level was measured using a commercially available kit from (R&D systems Kit, USA). The serum glutathione reductase activity was measured using a commercially available kit from (Biodiagnostic and research reagents, Egypt).

### Statistical Analysis

For descriptive statistics, the frequency and percentage were calculated for qualitative variables, the mean values ± standard error (SE) and range were used for quantitative variables. For comparison between the two groups, Student‘s *t*-test was used. For comparison between more than two groups, the ANOVA test was used. For correlation, Pearson correlation was used. For comparison of qualitative variables, the chi square test was used. If the assumptions of the chi square test were violated, Fisher’s exact correction was used. For the calculation of specificity and sensitivity of a given marker, the cut-off level is determined according to the following equation (cut-off level = mean of control +2 S.D). Statistical computations were done on a personal computer using the computer software SPSS version 13 (Chicago, IL, USA). Statistical significance was taken at P< 0.05.

## Results and Discussion

The characteristics of cirrhotic and HCC patients’ groups are summarized in Table 1, while tumor characteristics of HCC patients are given in Table 2. HCC, cirrhotic, and control groups were matched regarding age (P= 0.08).

**Fig. 1:**
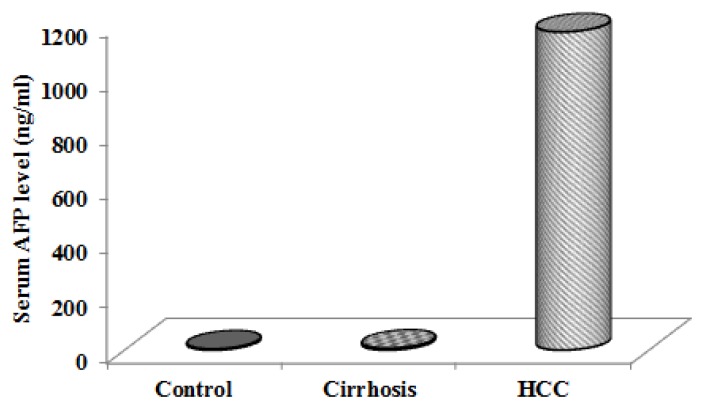
Serum α-fetoprotein (AFP) level in cirrhotic, hepatocellular carcinoma (HCC) patients and control group.

The distinction between HCC and cirrhosis has become challenging because regenerative nodules may mimic tumors in cirrhotic livers, and also because of elevated serum levels of AFP in patients with cirrhosis [11]. We found a significant increase in the serum concentration of α-fetoprotein in HCC patients as compared to patients with liver cirrhosis and the control group. Similar results were obtained by many studies [12–14] (Figure 1). Although AFP is a serological marker currently available for the detection of hepatocellular carcinoma, its poor sensitivity and specificity renders it unsatisfactory for this purpose and suggests the need for novel biomarkers for the detection of early HCC [15].

NO is a small, potent lipophilic gas with divergent biological activities that seems to play an important role in modulating tissue injury and carcinogenesis [16]. Serum nitrite/nitrate levels act as an index for *in viv*o NO production in patients with HCC [17]. Inducible nitric oxide synthase (iNOS) is related to a high output pathway for NO production which contributes to tumor cell angiogenesis as well as the invasion and metastasis of HCC [18]. Therefore, we aimed to measure the serum concentration NO in patients with liver cirrhosis and HCC to evaluate its activity as a tumor marker for liver malignancies.

**Fig. 2:**
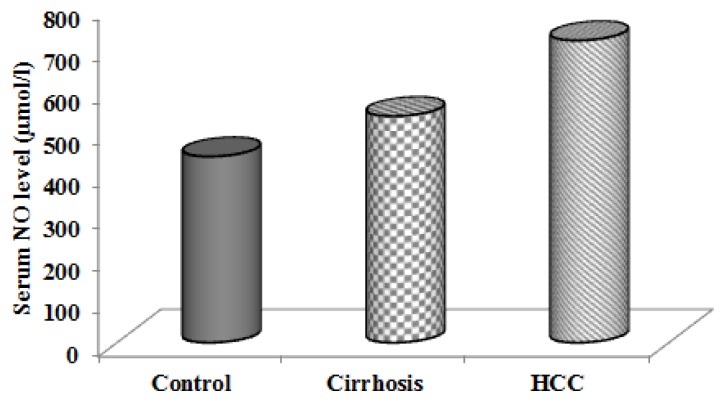
Serum nitric oxide level in cirrhotic, hepatocellular carcinoma (HCC) patients and control group.

Interestingly, as shown in Table 3, our study found that HCC patients showed significantly higher NO levels than patients with cirrhosis, and significantly higher than normal controls (Figure 2). This was in agreement with Mansurova, Osman, and Abd El Moety and Abd El Moety [19–21]. These data point to the potential value of nitric oxide in the diagnosis of HCC. As shown in Table 4, at a cut-off level of 200 ng/ml, AFP had a sensitivity of 52%, specificity of 100%, positive predictive value of 100%, and a negative predictive value of 65.2%. Other studies have shown similar results with the specificity of AFP close to 100%, but at a cost to the sensitivity which falls below 45% [22]. The diagnostic performance of nitric oxide in HCC patients at a cut-off of 614.1 μmol/l showed that the sensitivity, specificity, positive predictive value, and negative predictive value were (74%, 88.89%, 88.1%, and 75.5%, respectively).

As shown in Table 5, 24 out of 50 patients with HCC (48%) were AFP-negative. Notably, 62.5% (15/24) of these AFP-negative HCC patients were NO-positive. Thus, we found that the combined determination of AFP and nitric oxide had a sensitivity of 82% in the determination of HCC patients. Moreover, the simultaneous determinations of serum AFP and serum NO concentrations gave significant improvements in HCC detection compared with AFP alone and this was consistent with Moriyama [23]. Consequently, nitric oxide can be used as a complementary marker to serum AFP concentrations to detect HCC. However, due to the small sample size, these data need to be validated with a larger sample size.

Hepatocytes produce NO in response to several inflammatory stimuli. Tumor cells themselves are able to produce large amounts of NO due to induced expression of iNOS, which may prevail in rapidly growing tumors [24]. Thus, there is a possibility that NO production by hepatic tissue is accelerated in patients with HCC [17]. Increased NO generation is well-recognized as an essential step initiating neoplastic transformation [25]. Added to this, NO plays an important role in HCC development and its progression [26].

A possible explanation for increased serum NO levels in HCC is that NO is reactively induced by the hepatic tissue surrounding HCC by three independent mechanisms: (1) tumor cells directly stimulate macrophages and Kupffer cells to produce NO, (2) HCC produces a variety of cytokines that may stimulate hepatocytes to produce NO, and (3) a marked deterioration of liver function in HCC patients may be associated with increased portosystemic shunting and further development of hyperdynamic circulation, leading to an increase in NO production [6].

Our study found a significant positive correlation between serum nitric oxide levels and ascites in HCC patients (P= 0.026), which was in agreement with Mirodzhov [27]. On the other hand, there was an insignificant difference between serum NO in patients suffering from portal vein invasion compared to those without portal vein invasion. Consequently, no correlation was found between serum NO and an increasing stage of BCLC. No correlation between the concentrations of serum NO and serum AFP was found in patients with HCC and this was consistent with Moriyama [23]. In agreement with Moriyama [23], the assay for nitrate / nitrites is inexpensive and simple to perform; it may be a beneficial tool for screening HCC. In addition, the determination of nitrite / nitrate might be useful for the diagnosis of HCC.

Glutathione reductase is a homodimeric flavoprotein which catalyzes the reduction of oxidized glutathione (GSSG) to the reduced form (GSH) in the presence of NADPH as a reducing cofactor [28]. Glutathione reductase has a function in diverse cellular phenomena, including the defensive response against free radicals and reactive oxygen species (ROS), as well as protein and DNA biosyntheses via maintainence of a high ratio of GSH/GSSG [29].

**Fig. 3:**
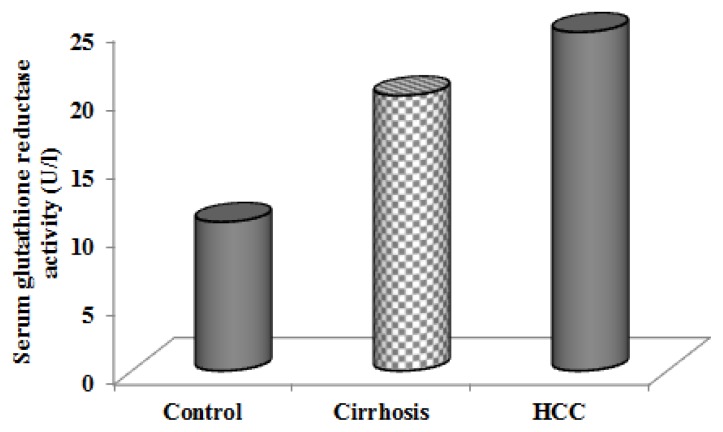
Serum glutathione reductase activity in cirrhotic, hepatocellular carcinoma (HCC) patients and control group.

As shown in Table 3, our findings showed that glutathione reductase activity was increased significantly in HCC patients with respect to the control (Figure 3). This was consistent with Czeczot [7], Scibior [30], and Tsai [31]. Also, the levels for liver cirrhosis tended to be higher than in controls, but the differences were not statistically significant.

The increased activity of glutathione reductase in the serum of patients with HCC may be a compensatory up-regulation [31]. Also, this increase in glutathione reductase is probably a function of the increased detoxification capacity as an adaptive response for oxidative stress [30].

Glutathione reductase was highly specific for GSSG and could be used in the detection of liver injury. So the increased activity of glutathione reductase could be explained by the fact that the increased production of reactive oxygen metabolites might be actively scavenged by GSH, resulting in the formation of GSSG, which is rapidly converted to GSH by glutathione reductase [8].

**Fig. 4:**
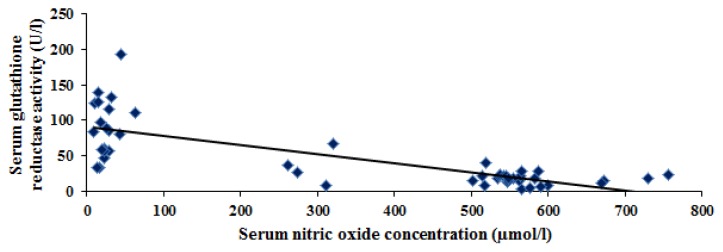
Significant negative correlation between serum nitric oxide concentration (μmol/l) and serum glutathione reductase activity (U/l) in cirrhotic patients (r= −0.424, p= 0.02).

A significant negative correlation was found between serum nitric oxide and glutathione reductase activity in cirrhotic patients (r= −0.424, p= 0.02) (Figure 4). *In vivo* inhibition of glutathione reductase activity by both exogenous and endogenous NO has also been reported [32]. Glutathione reductase maintains intracellular GSH levels and protects cells from oxidative stress and cytotoxic chemicals. Several reports suggest that RNS derived from NO decrease intracellular GSH levels [33]. Since glutathione reductase is responsible for keeping intracellular GSH levels constant, its inactivation by nitrosocompounds is a likely mechanism for this change [34]. This may explain the significant negative correlation between NO and glutathione reductase in cirrhotic patients.

**Fig. 5:**
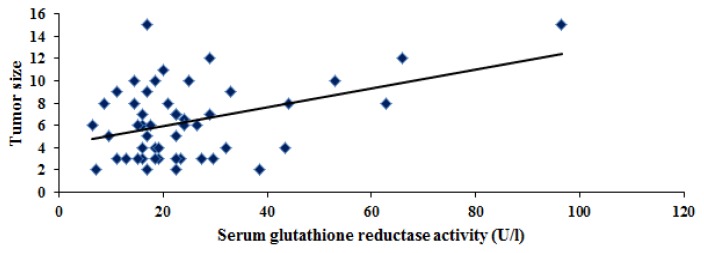
Significant positive correlation between serum glutathione reductase (U/l) and tumor size in HCC patients (r= 0.413, p= 0.003).

On the other hand, this study elucidated no correlation between serum glutathione reductase activity and serum AFP. However, a significant correlation between serum glutathione reductase activity and tumor size was found in HCC patients (r= 0.413, p= 0.003) (Figure 5). Further studies are needed to evaluate whether the presence of high levels of the antioxidant relieves tumors from oxidative stress allows them to reach larger sizes.

## Conclusion

This study demonstrated that serum nitric oxide and glutathione reductase levels are significantly elevated in patients with HCC. So we can conclude that:

1. Nitric oxide is a promising diagnostic marker for HCC that may aid in screening and detection of HCC.
2. The combined estimation of nitric oxide and AFP increases the sensitivity of detection and diagnosis of HCC to 82%.
3. Glutathione reductase is not a diagnostic marker for HCC. However, it correlates significantly with tumor size.
4. These findings should be validated in further studies recruiting a larger number of patients.

## Figures and Tables

**Tab. 1:** Characteristics of the studied patient groups

	HCC (n=50)	Cirrhosis (n=30)	P
**Sex**			

Male	37 (74%)	19 (63.33%)	0.3
Female	13 (26%)	11 (36.67%)	

**Child-Pugh classification**			

A	21(42%)	9 (30%)	0.4
B	18(36%)	11 (36.7%)	
C	11(22%)	10 (33.3%)	

**Ascitis**			

No	16 (32%)	14 (48.27%)	0.15
Yes	34 (68%)	15 (51.72%)	

n…number of patients.

**Tab. 2:** Tumor-related findings of the HCC patient group

	HCC (n=50)
**No. of lesions**	

Single	16 (32%)
Two lesions	15 (30%)
Three lesions	11 (22%)
Multifocal	8 (16%)

**Metastasis**	

Absent	31 (62%)
Present	19 (38%)

**BCLC**	

A	1 (2%)
B	17 (34%)
C	20 (40%)
D	12 (24%)

**Performance status**	

0	2 (4%)
1	22 (44%)
2	18 (36%)
3	8 (16%)

**Portal vein**	

Patent	37 (74%)
Thrombosed	13 (26%)

n…number of patients.

BCLC…Barcelona-Clinic Liver Cancer Group diagnostic and treatment strategy.

**Tab. 3:** Comparison of studied parameters in HCC vs. cirrhotic patients and control group (mean ± SE)

	Control (n=15)	Cirrhosis (n=30)	HCC (n=50)	P	Between group comparison	P
**AFP (ng/ml)**	4.17 ± 0.53	7 ± 2	1168 ± 307*#	0.004	HCC vs. Control HCC vs. Cirrhosis Cirrhosis vs. Control	<0.001 <0.001 0.12
**Nitric oxide (μmol/l)**	442.2 ± 29.06	537.96 ± 20.98	718.04 ± 36.53*#	<0.0001	HCC vs. control HCC vs. cirrhosis Cirrhosis vs. control	0.001 0.001 0.626
**Glutathione reductase (U/l)**	10.82 ± 0.99	20.01 ± 2.27	24.62 ± 2.3*	0.019*	HCC vs. control HCC vs. cirrhosis Cirrhosis vs. control	0.019 0.492 0.243

n…number of patients;

* …significant difference as compared with the control group at p< 0.05;

# …significant difference as compared with the cirrhotic group at p<0.05

**Tab. 4:** Sensitivity and specificity values for AFP and nitric oxide in HCC patients vs. cirrhotic patients and control group.

Markers	HCC (n=50)	Control and Cirrhosis (n=45)	Sensitivity	Specificity	+ve predictive	−ve value
AFP < 200	24	45	52%	100%	100%	65.2%
AFP > 200	26	0				
NO < 614.1	13	40				
NO > 614.1	37	5	74%	88.89%	88.1%	75.5%

n…number of patients.

**Tab. 5:** AFP and NO values in HCC patients.

AFP*	NO*		Total
	(NO > 614.1)	(NO < 614.1)	
**(AFP > 200)**	22	4	26
**(AFP < 200)**	15	9	24
**Total**	37	13	50

* … above and below the suggested diagnostic cutoff value.
